# Social influences on Moroccan and Pakistani immigrant women’s access and use of cervical cancer screening in Catalonia, Spain: a social network analysis

**DOI:** 10.1186/s12905-025-03657-8

**Published:** 2025-03-24

**Authors:** Jone G. Lurgain, Paula Peremiquel-Trillas, Hakima Ouaarab-Essadek, Khadija Mellouki, Andleed Sarif, Guy Harling

**Affiliations:** 1 Department of Public Health, Environments and Society, London School of Hygiene and Tropical Medicine, Keppel Street, London, WC1E 7HT UK; 2 Cancer Epidemiology Research Programme, Catalan Institute of Oncology, Av Gran Via 199-203, L’Hospitalet de Llobregat, Barcelona, 08908 Spain; 3 Bellvitge Biomedical Research Institute – IDIBELL, Av Gran Via 199-203, L’Hospitalet de Llobregat, Barcelona, 08908 Spain; 4 Consortium for Biomedical Research in Epidemiology and Public Health, CIBERESP, Carlos III Institute of Health, Av De Monforte de Lemos 5, Madrid, 28029 Spain; 5 Faculty of Medicine, University of Barcelona, C/Casanova, 143, Barcelona, 08036 Spain; 6 Community & Public Health Team (ESPIC), Centre for International Health and Infectious Diseases, Drassanes-Vall d’Hebron, Carrer de Sant Oleguer, 17, Barcelona, 08001 Spain; 7 Institute for Global Health, University College London, London, UK; 8 Africa Health Research Institute, KwaZulu-Natal, South Africa; 9 School of Nursing & Public Health, University of KwaZulu-Natal, Durban, South Africa; 10 MRC/Wits Rural Public Health & Health Transitions Research Unit, University of Witwatersrand, Johannesburg, South Africa

**Keywords:** Cervical cancer, Screening, Health inequities, Social influence, Behaviour change, Social network analysis, Migrant health

## Abstract

**Background:**

Participation in cervical cancer (CC) screening programs is lower among immigrants compared to native women in many Western countries, in substantial due to lower knowledge and culturally influenced attitudes regarding self-care and prevention. Education and information programs alone have limited impact on individuals’ attitudes and behaviours, but may be bolstered by social influence methods such as peer support.

**Methods:**

In this study, we combined self-reported quantitative structural social network data with qualitative narratives and graphs to describe the social context of 12 Moroccan and 10 Pakistani immigrant women living in Catalonia, Spain. We used a survey protocol and semi-structured interviews to explore how women’s contacts influence their CC screening behaviours.

**Results:**

We identified strong gender and ethnic homophily in these women’s social networks. Despite maintaining frequent remote contact with their family ties, their immigrant peers were more influential in providing health information and advice. Furthermore, the women’s husbands played two conflicting roles as health promoters and as a barrier to the use of health prevention services.

**Conclusion:**

Our findings highlight the need to incorporate tailored social influence approaches in the design of behaviour change interventions. In this case, the use of peer-based programs to increase CC screening uptake among these two immigrant communities.

**Supplementary Information:**

The online version contains supplementary material available at 10.1186/s12905-025-03657-8.

## Background

Although organised population-based screening programs are intended to reduce health inequities, numerous studies conducted in Western countries report lower cervical cancer (CC) screening uptake among immigrant women compared to native women [1–3]. This disparity increases the risk of delayed diagnosis and treatment and ultimately lower chance of survival in an already-disadvantaged group. Multiple barriers to CC screening have been identified across populations and settings, but the two most salient factors for immigrant women’s uptake of CC screening appear to be their limited knowledge about the disease, its causes and screening programs, and culturally influenced attitudes toward self-care and prevention [4–7]. Since increasing awareness through access to education and information does not alone change health beliefs, attitudes and behaviours [8], alternative approaches are needed.

One such alternative is the use of social influence methods to change individuals’ thoughts, feelings or actions through people or groups important to them [9]. The concept of social influence forms part of many theories and models of health-related behaviour, such as the theory of planned behaviour [13–14] and social norms theories [10–12], and has been used to guide preventive interventions, including CC screening. A key component of social influence on behaviour change relates to social norms. Social norms are unwritten and unspoken “rules and standards that are understood by members of a group, and that guide and/or constrain human behaviour” [10]. Social norm theory posits two types of norms: beliefs about what others will approve or disapprove of in a given situation (injunctive norms) and beliefs about what others do in a given situation (descriptive norms) [11–12]. Social norms about a health behaviour such as getting screened for CC – whether accurate or not – can act as a form of social influence if individuals adopt health behaviours based on either a perception that this is approved of by their social reference group (injunctive norms) [13–14] or the perceived prevalence of the behaviour among relevant peers (descriptive norms) [15].

Intention among women to participate in cancer screening programmes has been shown to be greater both with the perception that husbands, family, and close friends approve of screening and think they should do so (injunctive norms) [16–17] and when their close social contacts (e.g., sisters) were screened (descriptive norms) [18]. This means that individuals form opinions and beliefs about how they should behave through social interactions, observations, and information [9] and these social influence processes occur within individuals’ social networks.

Social networks provide the necessary structure for the transmission of knowledge and information, and the creation and diffusion of social norms that influence individuals’ behaviours [19]. According to social network theory, the structure of a network (e.g., composition) may also influence and predict health behaviours and outcomes [20]. An important distinction pertaining to network connections is that of strong and weak ties: the former are close relationships (i.e., with family and friends) and are important for emotional support, while the latter are more distant or casual relationships and are more valuable in terms of providing access to new information and opportunities [21]. For instance, Luque et al. (2016) [22] found that Peruvian Andean women with a higher proportion of weak ties, such as with neighbours, in their immediate network were significantly more likely to get screened for CC than those with a higher proportion of family and friends.

In conclusion, individuals’ health behaviours are likely to be influenced both by strong ties (e.g. family members) through injunctive and descriptive norms and weak ties (e.g. neighbours) or external information sources. Therefore, examining both social norms and social networks is crucial to deeply understanding social influence on health behaviours. Although several studies have explored the influence of social networks on immigrants’ health behaviours and outcomes [23–27], there is little evidence on how social networks may influence immigrant women’s self-care and prevention attitudes and behaviours. We therefore carried out a qualitative egocentric social network analysis (SNA) through interviews with immigrant women from Morocco and Pakistan living in Catalonia, Spain. The study is nested within the new organised population-based CC screening program implemented in this Spanish region and it aims to describe immigrant women’s social networks and explore how network members may influence their self-care and prevention attitudes and, particularly, their CC screening behaviour.

## Methods

### Study participants and settings

Participants in this study were women born in Morocco or Pakistan who migrated to Spain after the age of 16 (thus not exposed to Spanish public education and, therefore, less influenced by the Spanish culture) and have lived in Spain for at least one year. The Moroccan community is the largest group of foreign migrants in Catalonia (excluding Latin American immigrants), representing approximately 19% of the foreign population and 3% of the total population. Almost half of the Moroccan-origin residents in Catalonia are women [28], many of whom migrated to Spain through family reunification processes [29]. Many of them, especially first-generation immigrants, do not work outside the home; those who do work, often occupy precarious jobs (e.g., domestic work and hospitality industry) [29, 30]. The Pakistani community is small in Spain but is rapidly increasing: between 2010 and 2020 it increased by approximately 72%, with over half living in Catalonia, especially in and around Barcelona [28, 31]. Pakistani immigrant women comprise only 29% of this community [28] and mostly arrived through family reunification procedures predominantly sponsored by male family members (husband or father). Only a minority are active in the Spanish labor market [32].

Inclusion criteria for selecting the sample were the same as those used by the CC screening program in Catalonia: women aged 25 or who will turn 25 in the year of the study, to 65 who have never been diagnosed with this cancer. We used purposive sampling to get maximum variability based on different variables such as age, marital status, having children, length of stay in the country, host country language proficiency and employment status, and snowball sampling techniques. The recruitment process took place both in Barcelona city and neighbouring municipalities with high concentration of these two immigrant communities. The recruitment process and collected data were part of a broader implementation study of an organised population-based CC screening program in Catalonia, which included focus groups and semi-structured interviews. This is reported in greater detail elsewhere [33]. For this study, we only analysed data from the semi-structured interviews with 12 Moroccan and 10 Pakistani women. This sample size is comparable to other qualitative and mixed-methods egocentric network studies targeting ‘hard-to-reach’ populations [34–35] and allowed us to observe differences and similarities in the composition of participants’ social networks.

Sociodemographic characteristics of participants are shown in Table 1. Most women lived in industrial semi-urban areas with long history of migration although nine lived in Barcelona city. Women’s ages ranged from 24 to 65 years. All but three participants (all Moroccan) were married with children. Pakistani women were more educated on average, although education levels in both groups varied greatly. Moroccan women had lived longer in Spain on average, but the great majority of both groups had migrated over five years ago. Reflecting this longer residence, Moroccan women were more likely to be employed and to speak Spanish or Catalan at home. Most participants had received a Pap test in their lifetimes, but many had not had a Pap test in the past three years.

**Table 1:** Characteristics of participants (Egos)

Participants	Age	Married with children	Education	Employment	Time since migration to Spain	Host country language spoken at home	Religion (Muslim) self-identification	Number of years since last Pap smear
MC01	54	Married no children	Vocational training	Employed	32	No	Very little	2 to 3 years
MC02	41	Divorced with children	Primary school	Employed	26	No	Somewhat	2 to 3 years
MC03	26	Yes	Secondary school	Unemployed	8	No	Somewhat	3 to 5 years
MC04	38	Yes	University	Employed (informal)	16	No	Very religious	1 to 2 years
MC05	50	Yes	Secondary school	Unemployed	5	No	Somewhat	2 to 3 years
MC06	31	Single mother	Secondary school	Unemployed	6	No	Very little	Never screened
MC07	57	Yes	No formal education	Unemployed	20	No	Very religious	1 to 2 years
MC08	34	Yes	Vocational training	Unemployed	17	Yes	Somewhat	Never screened
MC09	24	Yes	Secondary school	Unemployed	3	Yes	Somewhat	1 to 2 years
MC10	65	Yes	No formal education	Unemployed	30	No	Somewhat	2 to 3 years
MC11	28	No	Secondary school	Employed	13	No	Somewhat	Never screened
MC12	45	Yes	University	Employed	13	No	Prefer not to answer	5 or more years
PC01	40	Yes	University	Employed	15	No	Somewhat	3 to 5 years
PC02	27	Yes	University	Unemployed	1	No	Somewhat	1 to 2 years
PC03	48	Yes	University	Unemployed	21	No	Somewhat	5 or more years
PC04	52	Yes	Secondary school	Unemployed	12	No	Very religious	3 to 5 years
PC05	29	Yes	University	Unemployed	7	Yes	Very religious	3 to 5 years
PC06	33	Yes	University	Unemployed	4	No	Somewhat	< 1 year
PC07	39	Yes	University	Unemployed	7	No	Somewhat	3 to 5 years
PC08	33	Yes	Secondary school	Unemployed	10	No	Very religious	1 to 2 years
PC11	57	Yes	No formal education	Unemployed	10	No	Very religious	Never screened
PC12	38	Yes	Primary school	Unemployed	9	No	Somewhat	3 to 5 years

MC = Morocco; PC = Pakistan

### Study design

To explore the social networks in which the participants were embedded and how these may influence their health behaviours, we conducted a qualitative egocentric SNA, combining structural network data (e.g. size, composition, density) with qualitative narrative accounts and graphs. SNA is defined as a set of methods used for mapping, measuring, and analysing social relationships, and it is uniquely suited to describing, exploring, and understanding structural and relational aspects of health [35–36]. Qualitative egocentric SNA, in particular, assesses the personal networks of an individual (‘ego’) across multiple social settings, by asking them to identify their network members -family, friends- referred to as ‘alters’ [37]. It also captures the meaning of these relationships and the extent to which these interactions can influence preferences, adherences to norms and decision-making [38–39].

### Social networks’ elicitation and alters’ attributes

We adapted the methods employed by Lubbers et al. (2007) [40] and Bidart et al. (2006, 2011) [41–42] in order to study immigrants’ and young people’s social networks in Spain and France, respectively. After initial questions about sociodemographic and CC screening status, participants were asked to name their immediate social network with no limit on the number of individuals they could nominate to estimate network size and capture strong and weak ties [43]. We used an interaction-based name generator adapted from Lubbers et al. (2007) [40]: ‘*Please list the people*,* family members*,* friends or acquaintances whom you know by name or by sight*,* and with whom you had some contact in the past two years either face-to-face*,* by phone or by the internet and whom you could still contact if you had to’*. Participants responded to the name generator sequentially for six contexts: family, friends, neighbourhood, leisure or educational activities, religious practice, and workplace, integrating the context-focused name generator approach used in Bidart et al.’s (2006) study [41].

Respondents provided information about their contacts’ attributes, e.g., sex, gender, country of origin, place of residence, and the nature of the ego-alter relationship. They were asked which contacts they would turn to for information and advice about CC screening, among other health-related questions; respondents could add individuals not already listed. They were also asked questions about social norms constructs, such as their female contacts’ CC screening status (descriptive norms) and the proportion of alters who ever recommended that they get screened (injunctive norms). Respondents ranked their alters by emotional closeness by placing them on a visual sociogram with three concentric circles: very close, close and distant. Finally, ego-perceived alter-alter ties were captured by drawing arrows between alters believed to know each other. Interviewers made notes of the women’s accounts about their interactions with their social contacts during the network elicitation (see SNA protocol in Supplementary material 1).

### Semi-structured interviews

Based on the visual sociogram, interviewers asked participants how their social networks had changed after migration and their perception of their new social networks in Spain. These questions served as a transition to a semi-structured interview (SSI) including questions to explore: (a) the role of family and friends on participants’ self-care attitudes and behaviours; (b) women’s access and use of health prevention services; (c) to what extent family and friends take part in women’s health decisions, especially about sexual and reproductive health (SRH) issues, and (d) their willingness to become ‘champions’ for CC prevention within their communities. These social influence questions were embedded into a broader SSI topic guide addressing barriers and facilitators to CC screening published elsewhere [44]. The topic guide was adjusted to explore emerging topics as data collection progressed. For example, we added a question to explore husbands’ objections to their wives visiting a male doctor (see interview topic guide in Supplementary material 2).

### Data collection and trainings

Data collection was conducted between September and December 2022. The computer-assisted personal interviews used Network Canvas [45], a social network-focused software that allows generation of quantitative measures, network visualizations and qualitative narratives [46]. Interviews were conducted either by the RAs (KM and AS) in Darija or Urdu, or by the first author (JGL) in Spanish. Interviews were conducted in private and quiet places, such as the participants’ own home when their children were at school, participants’ workplace, the mosque or interviewers’ homes. In some cases, the elicitation of the social network and the qualitative SSI were conducted on separate days due to participants’ busy schedules (e.g. childcare responsibilities). The interviews lasted between 1.5 and 2.5 h. All interviews were audio-recorded then translated and transcribed verbatim directly from the participants’ local languages into Spanish or English. The RAs (KM and AS) received three days of training in SNA methods, Network Canvas software and interview topics and techniques, and conducted debriefs with JGL after each interview to discuss challenges and identify emerging topics and patterns.

### Data analysis

We combined thematic content analysis with summary statistics and network graph comparison to first describe the social networks of these 22 immigrant women and, second understand how their social networks influence their self-care and prevention practices relating to SRH and CC. For qualitative content analysis, we followed the 6-phase guide proposed by Braun and Clarke (2006) [47]. We used a deductive approach and a constructivist lens to identify themes and sub-themes and develop a preliminary codebook. In this initial stage, the first author (JGL) and another researcher (PPT) hand-coded two initial interview transcripts and afterwards, they used Microsoft Office Word and Excel to define themes and subthemes. After consensus, the first author developed a final codebook using ATLAS.ti [48] and performed the content analysis. For SNA analysis, numeric data were cleaned and analyzed using Stata16 [49]. Network measures included network size (i.e., number of contacts listed), composition (i.e., proportion of network contacts who were female, immigrants, family members or were screened for CC) and density (the number of alter-alter ties present divided by all possible ties). We visualized each participant’s network including ego-alter and alter-alter ties using the igraph package [50] in R software [51] and we used them to highlight overall network patterns, differences between Moroccan and Pakistani women’s networks and other factors predicting network composition and structure.

### Ethical considerations

#### Ethical approval

was obtained from the institutions participating in the study: London School of Hygiene and Tropical Medicine (26186), Bellvitge University Hospital (PR 140/22) and Vall d’Hebron University Hospital (PR(AG)317/2022). Written informed consent was obtained from all the participants prior to data collection. Participants were given a public transportation 10-trip pass to acknowledge their contribution in the study.

## Results

### Moroccan and Pakistani immigrants’ social networks

#### Size and composition of participants’ networks

The 22 participants named a total of 485 social contacts, with an average of 22 contacts (range: 6–38) per participant, among both the Moroccan and Pakistani women. Social network composition was very similar in both groups, as shown in Table 2. Most of the contacts were female (*N* = 379, 78%) and were born in the participants’ country of origin (*N* = 367, 75%) or in third countries (*N* = 29, 6%), showing a strong gender and ethnic homophily (i.e., egos and alters were similar to one-another in these terms).

**Fig. 1:**
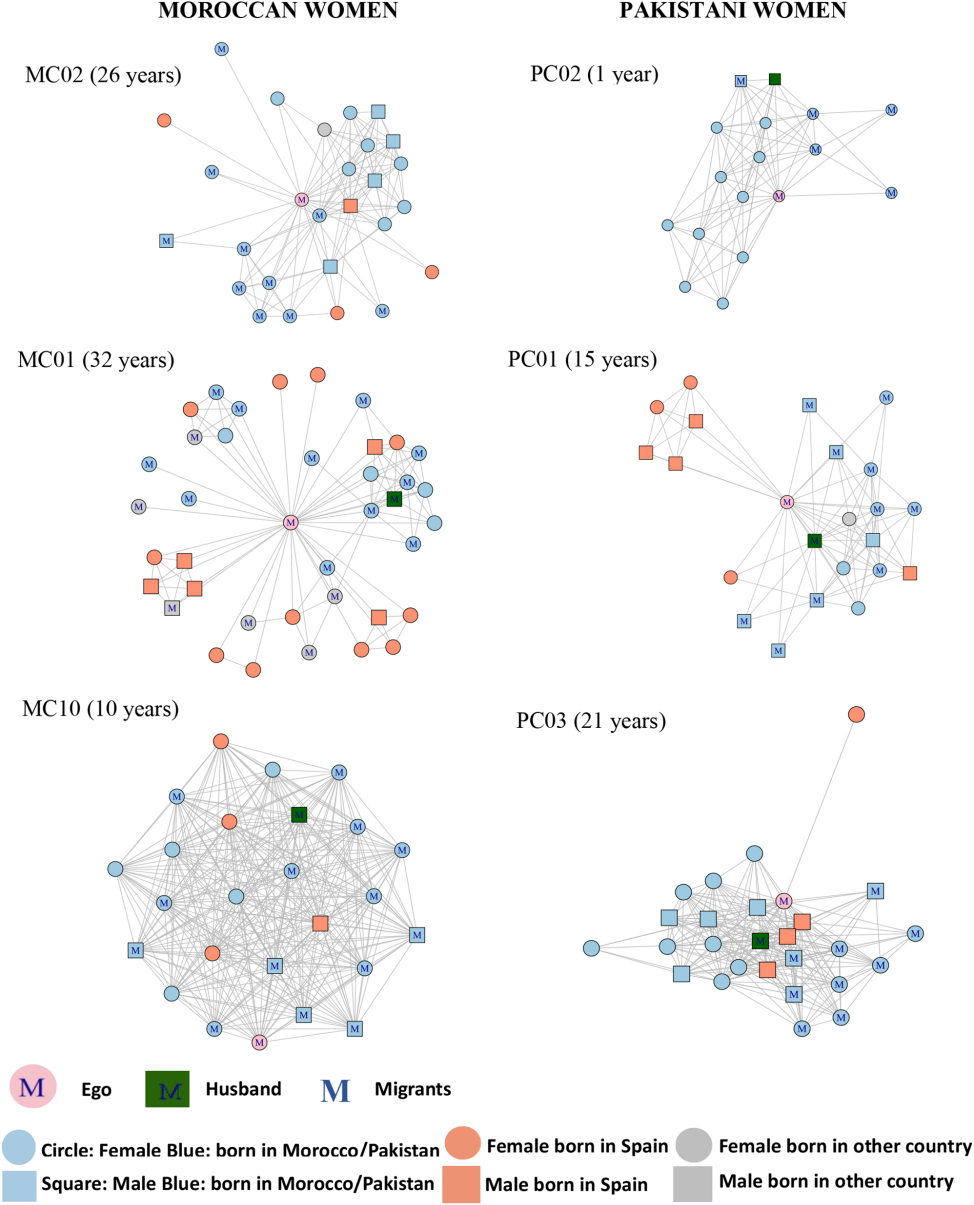
Examples of social networks of the study participants and length of stay in Spain. The top two network graphs illustrate the common tendency of gender and ethnic homophily in the Moroccan and Pakistani women’s social networks. The two graphs in the middle are examples of the social networks with the presence of job-related clusters of Spanish female and male contacts. The two graphs at the bottom show the high density of the women’s networks, based mainly on family members, all contacts know each other

Respondents had few contacts born in Spain (*N* = 84, 17%), as seen in their network graphs (Fig. 1), but Moroccan women had twice as many Spanish contacts as Pakistani women. This difference reflected Moroccan women’s greater integration in the Spanish labour market – all employed participants’ networks contained Spanish colleagues. In contrast, almost half of the contacts (*N* = 236, 49%) were close ties, such as family, and of these more than half (*N* = 135, 57%) were parents, siblings, and other close relatives (i.e., cousins and aunts) living in their home countries. Due to the distance of these transnational ties, husbands and in-laws seemed to play a central role in the participants’ new life in Spain, especially during the first years post-migration, providing them with guidance and companionship.

Friendship ties comprised around a quarter of all contacts (*N* = 124, 25%) and most of them (*N* = 91) living in Catalonia, reflecting the new relationships that women built in Spain and the significance of residential proximity for access to information and advice. Qualitative data highlighted that some close relationships reflected multiple roles: e.g., women considering their siblings, sisters-in-law, cousins, and husband as friends. Finally, women named almost as many weak ties (acquaintances) (*N* = 125, 26%) as friends (close ties) in their social networks. These weak ties were mainly women’s Spanish colleagues and neighbours, children’s teachers, health providers and immigrant peers.

#### Interactions’ patterns and connectedness between ties

Strong ties, such as husbands and children were the contacts with whom participants communicated most regularly (daily) and in person. Most interactions with acquaintances (e.g., colleagues, neighbours, teachers, other mothers in the park, health providers) were in person and quite regular (daily or weekly). As mentioned earlier, Moroccan and Pakistani participants seemed to speak with their parents (especially mothers), siblings and friends regularly but mainly through phone and online chat (Table 2).

Most participants had dense social networks (almost half of all possible alter-alter ties existed), which may be explained by the high proportion (almost 50%) of family members in the women’s networks. This was even more apparent in the two consanguineous marriages (of first cousins) as it is shown in Fig. 1 in the Moroccan participant MC10’s graph. Moroccan and Pakistani women mentioned an average of 9 strong or close ties (placed in the inner circle of the sociogram), representing nearly 42% of the total alters.

**Table 2:** Composition of social networks by Ego’s country of origin

		Moroccan women/Egos (*N* = 12)		Pakistani women/Egos (*N* = 10)		Total Egos (*N* = 22)	
		*N*	%*	*N*	%*	*N*	%*
**Total contacts (Alters)**		264	54.4	221	45.6	485	100.0
**Gender**							
	*Female*	211	*79.9*	168	*76.0*	379	*78.1*
**Age**							
	*≤ 29 years*	65	*24.6*	55	*24.9*	120	*24.7*
	*30–39 years*	62	*23.5*	74	*33.5*	136	*28.0*
	*40–49 years*	65	*24.6*	45	*20.4*	110	*22.7*
	*≥ 50 years*	72	*27.3*	47	*21.3*	119	*24.5*
**Education level**							
	*None*	40	*15.2*	9	*4.1*	49	*10.1*
	*Primary school*	34	*12.9*	12	*5.4*	46	*9.5*
	*Secondary school*	56	*21.2*	46	*20.8*	102	*21.0*
	*Vocational training*	22	*8.3*	22	*10.0*	44	*9.1*
	*University studies*	90	*34.1*	100	*45.2*	190	*39.2*
	*Koranic school*	4	*1.5*	2	*0.9*	6	*1.2*
	*Don’t know*	18	*6.8*	30	*13.6*	48	*9.9*
**Country of birth**							
	*Morocco/Pakistan*	188	*71.2*	179	*75.7*	367	*75.7*
	*Spain*	56	*21.2*	28	*17.3*	84	*17.3*
	*Other*	18	*6.8*	11	*6.0*	29	*6.0*
	*Don’t know*	2	*0.8*	3	*1.0*	5	*1.0*
**Country of residence**							
	*Morocco/Pakistan*	61	*23.1*	74	*33.5*	135	*27.8*
	*Spain*	188	*71.2*	129	*58.4*	317	*65.4*
	*Other*	14	*5.3*	18	*8.1*	32	*6.6*
	*Don’t know*	1	*0.4*	0	*0.0*	1	*0.2*
**Relationship of contact to respondent**							
	*Son/daughter*	20	*7.6*	13	*6.8*	33	*6.8*
	*Husband/partner*	8	*3.0*	10	*3.7*	18	*3.7*
	*Mother/father*	8	*3.0*	8	*3.3*	16	*3.3*
	*Siblings*	35	*13.3*	33	*14.00*	68	*14.0*
	*Other relatives* ^*1*^	40	*15.2*	24	*13.2*	64	*13.2*
	*Husband’s relatives*	17	*6.4*	20	*7.6*	37	*7.6*
	*Friends*	67	*25.4*	57	*25.6*	124	*25.6*
	*Acquaintances* ^*2*^	69	*26.1*	56	*25.8*	125	*25.8*
**Frequency of contact**							
	*Daily*	90	*34.1*	76	*34.4*	166	*34.2*
	*Weekly*	84	*31.8*	64	*29.0*	148	*30.5*
	*Monthly*	34	*12.9*	48	*21.7*	82	*16.9*
	*Less than once a month*	56	*21.2*	33	*14.9*	89	*18.4*
**Channel of communication**							
	*In person*	91	*34.5*	90	*37.3*	181	*37.3*
	*Phone*	43	*16.3*	41	*17.3*	84	*17.3*
	*WhatsApp or similar*	130	*49.2*	89	*45.2*	219	*45.2*
	*E-mail*	0	*0.0*	1	*0.2*	1	*0.2*

*Percentages correspond to row percentages. ^1^Other relatives refer to other women’s close relatives, such cousins, aunts. ^2^Acquaintances were colleagues, neighbours, immigrant peers/mothers, children’s teachers, health providers

#### Expansion of women’s social networks in Spain

Despite women’s narratives focusing on how their networks have decreased since migrating to Spain, both as a result of their distance from family and friends, but also because of their changing life circumstances (e.g., becoming wives and mothers for the first time), in fact, when asked to quantify and depict their social relationships, women reflected much wider social networks than they themselves initially perceived. The study participants explained how they built these new relationships in Spain, mostly with other female immigrants from their home countries. Since most women from both groups were housewives and mothers, their social interactions were mainly limited to the domestic sphere and public spaces where they took their children, e.g., schools and parks.

Another context where women had the opportunity to build new relationships was the mosque, suggesting also a potential homophilic tendency based on religion. Whereas most Pakistani participants reported attending social events at the mosque, only two Moroccan women reported participating in any activity organized at the mosque, despite most of them agreed that many women in their community often participate.

Participants’ new social networks in Catalonia, Spain, were also shaped by their personal characteristics (e.g., age) and circumstances (e.g., marital and employment status). For instance, having a job was an important determinant of women’s networks expansion. As this employed participant from Pakistan explained, her new network in Spain was more diverse than the one in her home country: *“I never thought that I could have male friends… For us*,* it’s a very big thing! We can have female friends but not male friends. Now I do have a male friend and I am happy and satisfied” (PC01).*

### The role of social networks on self-care and prevention behaviours

#### Normative influences on CC screening

Descriptive norms regarding CC screening differed between Moroccan and Pakistani participants: Moroccan women perceived that half of their female contacts had ever been screened, compared to one-third for Pakistani participants (Table 3). However, injunctive norms were very similar, with both groups believing that around one-third of the contacts would want them to get screened, and 15.9% of contacts had advised participants to get screened.

**Table 3:** Normative influences on cervical cancer screening by participant cohorts

	Moroccan women (*N* = 12)		Pakistani women (*N* = 10)		Total	
	*N*	%*	*N*	%*	*N*	%*
**Total Alters** ^**1**^	264	*54.4*	221	*45.5*	485	*100.0*
**Do you think this person has ever undertaken a Pap smear?** ^**2**^						
***Yes***	105	*49.7*	58	*34.5*	215	*44.3*
**Do you think this person thinks you should undertake a Pap smear?**						
***Yes***	96	*36.4*	65	*33.2*	161	*33.2*
**Has ever this person recommended you undertaking a Pap smear?**						
***Yes***	42	*15.9*	30	*13.6*	72	*14.8*

*Percentages correspond to column percentages. ^**1**^Percentages correspond to row percentages. ^**2**^Percentages were calculated only among female contacts

#### Female family and immigrant peers

Data from the qualitative interviews confirmed the gender and ethnic homophily identified in the egocentric network analysis. Most women highly valued the consultation and advice of close ties for health-related decisions, particularly in the context of self-care and prevention practices. They expressed a special trust and comfort in discussing health matters with other female close contacts, although their preferences for information and advice networks varied depending on the health topic. For instance, participants in both groups identified family members as the primary source of encouragement for self-care practices, such as exercise or maintaining a healthy diet. Nonetheless, when dealing with more sensitive and intimate health issues, such as gynaecological problems, it seemed that women preferred to seek advice from their immigrant peers, meaning those new ‘friendships’ formed with other women in their children’s school, parks, mosques, and other public spaces since their arrival in Catalonia (weak ties), including sometimes in-laws and other relatives. One Pakistani woman explained that they always avoid causing worry to their families (especially, mothers) back home about their health problems, and that they find it challenging to discuss women’s health issues with male family members due to social taboos. Moroccan and Pakistani participants describe below what type of health information, advice, and experiences they often share with their immigrant peers:

*“Most of my friends here are my age and we have similar health issues and experiences. We share our intimate things (…) I trust them*,* they are friends. These intimate things cannot be shared with all people. And with men*,* never! Because I can’t ask men for advice about the female reproductive system” (PC03)*.

*“We always talk about our things. She [her friend] also has itching (in her genitals) and me too… I remember the last time we were together; she told me that the itching is due to a bacterium that her husband got and she told me that one day she asked her husband: “are you going to meet someone tonight?” And he said: “no”. She told me that she was scared of men*,* because they go out a lot and she was afraid of men coming back home with a disease. So I tried to calm her and told her not to be worried” (MC04)*.

*“One friend told me ‘But where do you live? I have put an IUD ‘down there’ and every year I do a check-up’. She told me that she didn’t have her period for two months and when I told her about this cervical cancer*,* she was afraid of it” (PC05)*.

Interestingly, when women were asked who would they ask for advice before undergoing CC screening, they mentioned health providers first (e.g., doctors, nurses, pharmacists, medical students). Noteworthy, many of these professionals were relatives belonging to their immediate social networks (strong ties): *“I always ask my auntie because she is a doctor in Morocco and she knows about all these things [gynecological check-ups]” (MC11).*

On the other hand, the vast majority of women highly valued the advice and experience of other female immigrant peers regarding health prevention services in Catalonia, especially those women who had been living in Spain for a longer time, and those who had been married and had children. Particularly, when women were asked from whom they would seek help to use a HPV self-sampling device, they, once again emphasized the importance of learning from experienced peers who had already used the device and were familiar with navigating the Catalan health system.

*“I would ask my sister-in-law about cervical screening and this test [HPV self-sampling] because she has done it before and has experience about it” (PC06)*. *“I would ask advice from my cousin and also from my husband’s sister and my mother-in-law*,* because these people live here and they understand better and can guide me better than my family in Pakistan; even if they are health workers*,* I prefer people who live here (Spain)” (PC12)*.

Finally, most participants from both groups expressed their willingness to become ‘champions’ of CC prevention, encouraging and helping other women to use HPV self-sampling devices: *“In our communities*,* we always pass valuable information to others*,* if one person knows about this cancer test*,* she will tell about it to another and this one to another one*,* and so on” (MC04).*

#### The role of the husband

The multiple roles of the husband in the lives of Moroccan and Pakistani participants in Catalonia, Spain, were present implicitly and explicitly throughout the qualitative interviews. Particularly, we identified two conflicting influences of the husband: as a health promoter and as a barrier to health service use.

Participants in both groups highlighted the emotional and economic support, as well as the guidance provided by their male partners (strong ties) during the first years after migrating, particularly to navigate the Catalan health system: *“My husband used to come with me to the doctor*,* he taught me where was everything*,* what I had to do to go alone to the doctor… He was the only one who supported me in the beginning” (MC03).* Participants also emphasized how their husbands encouraged them to self-care and access health prevention services:

*“When I’m tired or I don’t feel well*,* my husband tells me directly to do a check-up. He always tells me: “If you don’t make the appointment*,* I’ll do it for you”*,* then I do it myself” (MC04)*.

*“He always tells me that I need to do regular check-ups*,* because he always says that we need to know whether we have a disease asap*,* so that we can seek a solution*,* or the doctor can tell us how we need to take care of ourselves or what we can do” (PC03).*

Although the above narratives show a supportive role of husbands concerning their wives’ overall health, male partners appeared poorly engaged in women’s SRH issues, except when they were pregnant, or the couple faced fertility problems. Some women explained that these topics are not shared with males in their cultures, but, paradoxically, in our study we identified an important limiting role of some husbands who seemed to prevent women from access and use of gynaecological preventive health services, for instance, not giving them permission to visit a male doctor, as these participants’ accounts show:

*“Someone told me that his wife didn’t visit the gynaecologist until her waters broke. When she went to the hospital here in Terrassa her husband asked for a female doctor to attend his wife (…) and the doctor told him that there was not a female doctor in that moment*,* but he insisted: ‘she’s my wife and I don’t want a man to help my wife to give birth’. Eventually*,* the husband was asked to leave the hospital and doctors attended the woman” (MC05)*.

*“It can be the case*,* but these are men from rural areas with low education*,* they think ‘if a man touches my wife*,* it will be sinful’” (PC07)*.

*“There are cases… there was a woman in my neighbourhood in Morocco who didn’t go to any check-ups*,* the doctor had to be a female*,* otherwise she didn’t go… It may be the case that her husband didn’t allow her to go” (MC06)*.

Finally, we observed different levels of autonomy in terms of SRH decision-making between Moroccan and Pakistani women. Whereas most Pakistani participants reported making all their health decisions together with their partners. Moroccan participants considered women’s health issues as personal and many of them emphasized that the decision on whether or not to get screened for CC was theirs to make. The below excerpts illustrate this:

*“I consult everything with my husband” (PC07)*.

*“I wouldn’t ask for advice to anyone*,* it’s not necessary. I’d just inform them (family) that I got an appointment with my gynecologist*,* that’s it” (MC12)*.

## Discussion

In this study we examined how the transmission of advice and social norms within Moroccan and Pakistani immigrant women’s social networks in Catalonia, Spain affects their preventive health behaviours, especially regarding CC screening. First, we found that female immigrants from the participants’ country of origin dominate Moroccan and Pakistani women’s social networks. Second, although frequent remote contact with their close family ties was reported, women’s most influential reference groups for SRH matters were weak ties, such their immigrant peers in Spain, including in-laws and other relatives. Third, despite female contacts dominating the core information and advice participants’ networks, husbands play a prominent role in facilitating or constraining women’s access and use of health services, including CC screening.

The strong gender and ethnic homophily seen in these Moroccan and Pakistani women’s social networks is consistent with previous research conducted with different immigrant populations [52–55]. This homophily influences the information these women receive from others, as well as their attitudes and health-related decision-making, and may arise from at least two sources. First, community social norms, such as sex segregation and gender roles of women (i.e., mothers, housewives) shape participants’ social interactions and the composition of their social networks. Notably, our findings show how participants’ new relationships were mainly developed at children’s schools, in parks and mosques (where sex segregation is the norm). Second, ethnic homophily may reflect the length of stay and proficiency in the majority language in the host country [56–57]. However, our study does not support this evidence, as most participants show a strong ethnic homophily despite having high Spanish language proficiency and living for a long time in Spain. An alternative explanation in these groups may be proximity (we are more likely to have contact with those who are closer to us), given the high concentration of immigrants in specific residential areas of Barcelona province. This hypothesis aligns with evidence from Scotland that movers tend to be drawn to areas with higher concentrations of ethnicities similar to their own [58].

Moroccan and Pakistani women mentioned an average of 9 close ties, representing nearly 42% of the total contacts, which may respond to the cultural context. Family relationships are the most important component of social life in Moroccan and Pakistani societies, where people often live in multigenerational households and, therefore, have social networks mainly composed of close family ties. Respondents valued consultation with their families back home before making a health decision. Nonetheless, for more private issues related to SRH, participants reported a high degree of trust in weak ties, such as their female immigrant peers, reflecting the above ethnic homophily and the strength of weak ties [21].

Women highlighted the importance of learning from those peers with more experience in Spain and better understanding of the Catalan health system and particularly the CC prevention services. This exchange of health knowledge between immigrant women was also reflected in a study conducted in USA with women from Guatemala [59]. Contrary to other migrant studies in Sweden and China [60, 27] which associated ethnic homophily with inadequate access and use of health information and services, in our study we identified an opportunity to leverage newly formed, but still ethnically homophilic, peer connections to replace older normative views about preventive health behaviours with new ones. These peer connections may definitely improve access and use of healthcare services, including CC screening uptake. This approach is aligned with recent implementation studies conducted in Uganda, Canada, and USA, in which researchers used peer-based interventions to promote and increase uptake of CC screening [61–63]. In our study, most participants expressed their willingness to become ‘champions’ of CC prevention, suggesting that word-of-mouth communication may also be crucial for information transmission in these two communities, especially to reach those women more isolated.

The important role that the male partners play in women’s health decision-making could override the opportunity that these immigrant peers offer to the participants to access new health information and dissemination of adapted norms around screening, with beneficial (if the husband is particularly supportive of preventive health behaviours) or negative effects (when the husband poses barriers to accessing health care). For instance, participants emphasized the husbands’ role as health promoters providing examples in which they encouraged women to self-care to prevent or manage chronic conditions. This health promoter role was not so obvious, however, when it came to SRH matters, which echoes the lack of engagement of husbands in CC prevention among Muslim families in Indonesia [64]. On the other hand, women reported the constraining role that conservative male partners can play in not giving their wives permission to visit a male doctor. This important barrier to women’s access and use of health services has also been identified in other studies conducted recently on CC screening uptake among under-screened women [61, 65–66]. To date, a growing body of studies conducted mainly in low- and middle-income countries has explored the potential engagement of husbands in CC screening interventions aiming to increase women’s uptake [67–70]. However, further research is needed to better understand male partners’ social norms around spousal support for preventive health behaviours and to evaluate the inclusion of men in CC screening interventions, especially in Western countries.

We also explored potential influences of injunctive and descriptive norms on CC screening behaviours. Only 15% of contacts had ever recommended women participants to get screened and this percentage corresponds mainly to siblings and immigrant peers, which reinforces their potential influence on CC screening uptake. On the other hand, while Moroccan participants believed that 50% of their female contacts had been screened, Pakistani women believed that only 34% of their female contacts had done so, which could be linked to the different levels of autonomy regarding SRH decision-making shown between Moroccan and Pakistani participants.

In sum, our findings point to how immigrant women’s experience of social influences and how their sources of information and advice change over time. Women rely heavily on strong ties (i.e. husbands) when they first move and settle into Spain, but over time, often without noticing, many participants expanded their social networks and created trusting relationships with other female immigrants peers (weak ties). This had the impact of expanding support sources for SRH matters beyond the husbands’ sphere to also include their immigrant peers in Spain. There are changes across time and space that are relevant to engaging with the health system, and therefore, possible ways to leverage those new relationships and connections for positive change in relation to screening behaviours among immigrant populations and, particularly, Moroccan and Pakistani women living in Catalonia. For instance, engaging those immigrant peers with longer exposure to and more experience with the Catalan health system in the transmission of health information and new norms related to preventive health behaviours, in this case, to increase CC screening uptake, would be an effective approach.

The study presents several strengths alongside with certain limitations. The most salient strength is the combination of multiple research methods and the triangulation of data: egocentric network measures, graphs, and narrative accounts. This comprehensive approach offers a greater depth of understanding regarding the social networks within which Moroccan and Pakistani immigrant women are embedded, shedding light on the potential influences of these social relationships on women’s health behaviours. In terms of limitations, the sample size of 22 immigrant women restricts the measurement of the effect of informational and normative influences on participants’ behaviour, a consideration beyond the scope of this egocentric formative research. Nevertheless, it establishes a foundation for future survey studies. Moreover, the theoretical lens (social norms and social network theories) used for this study will have influenced our findings and other theories, such as Moscovici’s social representation theory, could be a useful additional tool in future research. Additionally, the adoption of a maximum variability sampling approach allowed for meaningful comparisons between women’s social network patterns. Also, the use of multiple languages (Spanish, Darija-Arabic, Urdu) might have influenced the elicitation of different social networks. In the anticipation of this issue, the two bilingual RAs underwent comprehensive training to ensure consistent understanding of question meanings across languages. Furthermore, using all three languages for interviews facilitated outreach to a wider spectrum of immigrant women, including those proficient in Spanish. Finally, acknowledging the potential respondent burden in social network studies, efforts were made to minimize participants’ fatigue and avoid losing their attention and motivation, which affects the quality of data [71]. To mitigate this, the protocol was kept as shorter and concise as possible, incorporating breaks, and, in some cases, conducting the SNA data collection and the qualitative interview on separate days to ensure data quality.

## Conclusions

This study provides valuable evidence on the need of social influence approaches to behaviour change for the effective design of health promotion interventions. Particularly, in the context of the new Catalan organised population-based CC screening program implementation, this study emphasizes the imperative of leveraging the social networks of migrant and other underserved populations. The strategic utilization of social networks becomes crucial not only for disseminating accurate information on CC prevention, but also to foster a shift in perceptions and health prevention behaviours within these communities. Furthermore, the study identifies a significant research gap related to the potential role of male partners in enhancing women’s CC screening uptake. A deeper understanding of men’s social norms concerning the support of women’s preventive health behaviours is needed. Such insight will offer an opportunity to assess the feasibility to incorporate male partners into future CC screening interventions in Catalonia, Spain, and beyond. This holistic study acknowledges the interconnectedness of social networks and the need for inclusive strategies in promoting women’s health within immigrant communities.

## Electronic supplementary material

Below is the link to the electronic supplementary material.

Supplementary Material 1

Supplementary Material 2

## Data Availability

The datasets generated and/or analysed during the current study are not publicly available due to maintaining the privacy and confidentiality of participants, but are available from the corresponding author upon reasonable request.

## Abbreviations

CC: Cervical Cancer
HPV: Human Papillomavirus
IUD: Intrauterine Device
RA: Research Assistant
SNA: Social Network Analysis
SRH: Sexual and Reproductive Health
SSI: Semi-Structured Interviews
STI: Sexually Transmitted Infections
