# Supplementary material for: Social influences on Moroccan and Pakistani immigrant women’s access and use of cervical cancer screening in Catalonia, Spain: a social network analysis
Source: BMC Womens Health. 2025 Mar 24;25:136. doi: 10.1186/s12905-025-03657-8 (PMC11931830; doi:10.1186/s12905-025-03657-8)
Supplement: Supplementary file 1 — Supplementary Material 1 [file 12905_2025_3657_MOESM1_ESM.pdf]

**Protocol for social network data collection (Network Canvas)**

**Participant ID: ……………………………………………………………………………..................**

**Interview place: ……………………………………………………………………………….**

**Researcher and/or interviewer ………………………………………………………………**

## 1. Questions about Ego (participant)

***Thank you for giving your consent to be interviewed. I would like to start this interview asking you some personal questions.***

1.1. What is your date of birth? dd / mm / yyyy
1.2. What year did you migrate to Spain?
1.3. How many people do you live with? I live alone / 1 / 2 / 3 / 4/ 5 or more / Prefer not to answer
1.4. Which language(s) do you speak most often at home? (you can select more than one)
Spanish / Catalan / Arabic-Darija / Urdu / English / French / Other (specify) / Prefer not to answer
1.5. What is your marital status? Single / Married or living with my partner / Separated / Divorced/ Widowed / Other (specify) / Prefer not to answer
1.6.Do you have children? Yes / No / Prefer not to answer
1.7. If so, how many children do you have? 1 / 2 / 3 / 4 / 5 or more / Prefer not to answer
1.8. Tick the boxes in relation to your education (you can tick more than one):
No academic education / Primary school / Secondary school / University studies / Vocational training / Koranic school / Other (specify) / Prefer not to answer
1.9. Are you employed?
Yes, I am employed (full-time, part-time, self-employed) / No, I am unemployed (housewife) / Informal employment / Retired / Prefer not to answer
1.10. Can you give an estimate of your annual household income?
< €12,000 / € 12,001 – €20,000 / € 20,001 – 30,000 / € 30,001 – 40,000 / > €40,001 / I don´t know / Prefer not to answer
1.11. Do you have public health insurance in Catalonia? Yes / No / Prefer not to answer
1.12. If you have ever attended a gynaecologist appointment, have you ever undertaken a cytology?
Yes / No / Don´t know / Prefer not to answer
1.13. If you have ever undertaken a cytology, how long is it since the last time that you did so?
Less than 1 year / between 1 and 3 years / between 3 and 5 years / for more than 5 years / I don´t know / Prefer not to answer
1.14. Would you say you are a religious person?
Very religious / Somewhat religious / Very little / No religious at all / Prefer not to answer
1.15. Do you identify yourself as a Muslim person? Yes / No / Prefer not to answer

## 2.Name Generator I – Personal Networks

***Now we are going to map your personal network and we would like to ask you to list the people with whom you interact regularly in your life. You don't need to give me their names, you can use their initials or a nickname, if you prefer. If two persons have the same name we will need to use their surname initials or distinguish them in another way.***

Please, list people whom you know by name or by sight, and with whom you had some contact in the past 2 years either face-to-face, by phone, of by the Internet and whom you could still contact if you had to, in the following social contexts of your life.

***Per each question there may be a few people or none that fit the description. You can mention the same person more than once, in case the person fit more than one description. There is not a correct number of people per each question.***

2.1.We can start by your family circle. These can be your own family or your spouse's family members, and they can live either in Barcelona, other Spanish region or abroad.
2.2. Let's continue with your friends. Do you have any friend or friends to whom you are most often in contact (they can live in Spain or abroad).
2.3.Think about your neighborhood. Who are the people with whom you talk a little more and you can contact if you need to? For example, when you go to the shop, to the "*casal*" or your children's school.
2.4.Let´s talk about your religious practice, do you go to any religious center in Barcelona? In this context, do you have any person who is more special or closer to you?
2.5. In your free time, do you participate in any educational or leisure activity, such as language classes, sewing or you are a member of an association? Do you have any person with whom you like to spend more time in these activities?
2.6.Let's think about your workplace. Is there anyone in this context you know a little better, with whom you talk a little more or you used to work with and you are still in contact?

## 3.Name Generator II – Health information and advice networks

***Now I will ask you questions about the people with whom you talk about health. These may be people you already mentioned or other people, such as your doctor, a intercultural mediator or someone who lives abroad.***

3.1. Whom would you turn to for information and advice about an important problem or decision related to women´s health, such as having children? (Why these people?)
3.2. Whom would you turn to for information and advice about an important problem or decision related to women´s health, such as symptoms of a STI? (Why these people?)
3.3. Whom would you turn to for information and advice to decide whether or not to undertake a cytology or get screened for cervical cancer? (Why these people?)
3.4. Whom would you turn to for information and advice about how to use a new device to collect yourself a vaginal sample at home for cervical cancer screening? (Why these people?)
3.5. Is there any other relevant person that you didn´t mention?

## 4.Alters´ attributes and composition of personal networks

***I will ask you now some personal questions about the people you mentioned and the type of relationship you have with them.***

4.1. Is this person female or male? Yes/No
4.2. What type of relationship do you have with this person?
Sons-daughters / Husband-Partner / Father-Mother / Sister-Brother / Other close relatives / My husband-partner´s family / Friend / Acquaintance (specify)
4.3. What is the education of this person? No School / Primary school / Secondary school / Vocational training / University degree / Koranic school / Other (specify)

4.4. In which age range is this person? Less than 30 years / between 31 and 40 years / between 41 and 50 years / less than 50 years
4.5. Where is this person from?
From the country I was born / From Spain / From another country / I don't know
4.6. Where does this person live?
In the country I was born / In Spain / In another country / I don't know
4.7. How often do you talk with this person?
Daily / weekly / monthly / less than once a month
4.8. How do you usually communicate with this person?
Face to face / phone / WhatsApp or similar / email / letter

## 5.Conversations about cancer and cervical cancer screening

***Now I will ask you questions about the people to whom you have ever talked about cancer related topics.***

5.1. Have you ever talked about cancer with this person? Yes/No
5.2. Do you think this person think you should undertake a cytology or get screened for cervical cancer? Yes/No *(*If you are not sure or you don't know the answer, select the option 'NO')*
5.3. Have this person ever recommended you to undertake a cytology or get screened for cervical cancer? Yes/No

## 6.Female contacts´ cervical screening status

***Now, I will ask you only about your female contacts and cervical cancer screening.***

6.1. Do you think this person has ever undertaken a cytology or cervical cancer screening? Yes/No *(*If you are not sure or you don't know the answer, select the option 'NO')*

## 7.Strength of relationships and links between contacts (Sociogram)

***Now, I am going to place all the people you mentioned on a map called sociogram. This is a sociogram. You are the central point and the cycles surrounded you represent the people who are part of your social network or you interact with in your life. They may or may not be close to you (emotionally speaking).***

1. ***The inner circle is for "people to whom you feel so close that it is hard to imagine life without them" (for example, for me it would be my mum).***
2. ***The middle circle is for "people to whom you may not feel quite that close but who are still important to you" (for example, for me it could be a close friend).***
3. ***And finally, the third outer circle is for "people who are not so close emotionally, but they are somehow relevant enough to be in your social network" (for example, for me it could be a workmate or my GP).***

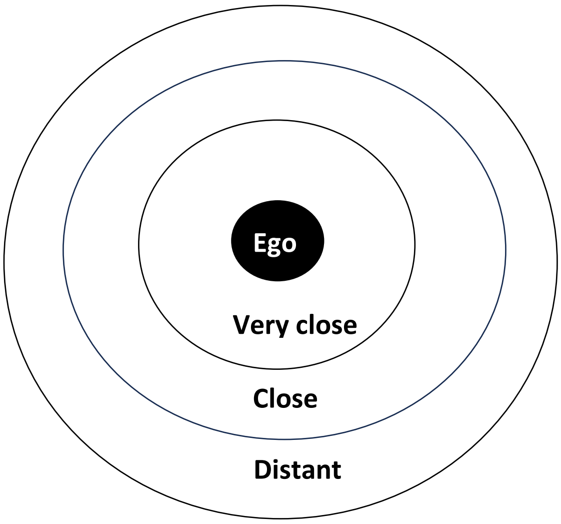


7.1. Please, place each person of your social network in the corresponding circle according to how close emotionally you feel towards them.

***To end, I will ask you about the connections between the people you mentioned.***

7.2. Please, can you add an arrow between the people whom know each other. By “know each other” we mean that they might contact, meet or talk to each other even if you are not there.
