# Supplementary material for: Social influences on Moroccan and Pakistani immigrant women’s access and use of cervical cancer screening in Catalonia, Spain: a social network analysis
Source: BMC Womens Health. 2025 Mar 24;25:136. doi: 10.1186/s12905-025-03657-8 (PMC11931830; doi:10.1186/s12905-025-03657-8)
Supplement: Supplementary file 2 — Supplementary Material 2 [file 12905_2025_3657_MOESM2_ESM.pdf]

Semi-structured interview topic guide with Moroccan and Pakistani women

Participant ID: ………

Participant place of residence: …………………………………………………………………………………

Interview place: ……………………………………………………………………………………………………….

Researcher and/or interviewer ……………………………………………………………………………………..

MIGRATION, SOCIAL SUPPORT AND SOCIAL NETWORKS

With your social network map in front of us, I will ask you some more questions about your contacts and health.

1. How does change your network of contacts before and after migrating to Spain? For example, is your current social network very different than your social network back in your country?
2. Looking at your contacts in the sociogram, who are the most supportive people in your life?

BARRIERS TO ACCESS HEALTH CARE SERVICES AND HEALTH SEEKING-BEHAVIOURS

Let's continue talking about your health.

3. When and why do you usually go to the doctor?

Prompts

-Do you go to the doctor as frequently as you did in your country?

-What do you think about the health system here? How are the doctors?

-For example, when do you go to the emergency services? Only when you feel very sick?

-Do you usually use traditional medicine from your country before going to the doctor? Give me details.

4. What are the main barriers you confront when you need to access health services?

HEALTH PREVENTION AND SOCIAL SUPPORT NETWORKS (e.g. social norms)

5. How much would you say you self-care and what do you do to take care your health?
6. How much do your family, for example, your husband or daughters, care about your health? How supportive are they? Give me some examples, please.
7. What kind of recommendations do your family and friends give you about health in general? And in particular, do they encourage you to do regular check-ups?

Prompts

Do they give you religious or spiritual advice?

Do you keep healthy diet, exercise, etc.?

8. Why do you think people do health regular check-ups?
9. In your country, do people do regular health check-ups?
10. If you would know that some diseases have not symptoms, would you do check-ups more regularly?
11. What do you think about doing tests to detect early a disease, like cancer? Are they useful, do you think they can prevent cancer?
12. Wha do you think your family and friends think about prevention programmes?

CONVERSATIONS ABOUT CANCER AND CERVICAL CANCER (awareness and knowledge)

Let's look at your social network again.

13. You said that you talked about cancer with a few people (question 5.1.) How do you think your family and friends feel when they hear the word 'cancer'?
14. Do you and the people in your family and friend circles talk naturally about cancer or you try to avoid this kind of conversations? Why?
15. Is there anyone in your family and friends circles whom has ever experienced cancer?

   YES – NO

<u>Prompts</u>

If yes, please, could you tell how many and how did you know about it?

Let me know a little bit more about these cases. How did you support them?

16. Before we said that a 'cytology' test is to detect cervical cancer. Is there any woman in your family or friend circles whom has ever experienced cervical cancer? YES – NO

<u>Prompts</u>

How many? Can you share your experience? How did you know? Did they share it with other people? What was your reaction? How did you support them?

CERVICAL CANCER SCREENING AND SOCIAL SUPPORT NETWORKS (barriers/facilitators)

Let's focus on women´s health issues.

17. Before you said that you have/have never got screened for cervical cancer o undertaken a cytology (question 1.12 and 1.13),
   - If NOT or LONG TIME AGO, why?
   - If YES, did you got screened in Barcelona and/or your country? <u>Tell me about your experience.</u>

<u>Prompts</u>

Whom did/does arrange the appointment with the gynaecologist for you?

What was the reason of the visit(s)?

Did you need a translator? With whom did you go?

How do you remember the last appointment? Was the doctor female or male? How supportive, sensitive he/she was?, was it painful, embarrassing?

Is there any difference between here and your country?

18. If you need help with the Spanish language, whom you prefer to go with or assist you when you go to a gynaecologist appointment,? Why?
19. Has an intercultural mediator ever helped you during a gynaecologist appointment? How was it? (e.g. helpful, do you prefer someone else?)
20. Does your family, for example, you husband has a say in decisions that affect your health and, in particular, your sexual and reproductive health? Let me know how do they get involved in the decision-making.
21. Whom did you influence or would influence you to undertake a cytology or get screened for cervical cancer? Why these people's opinion influence you and not others?

Prompts

Do they tell you what you should do?

Do they make decision for you?

22. Do you know any woman in your community who needs to ask permission to her husband to go to the doctor, to the gynaecologist? Could you share the case with me, please?
23. Why do you think women like you might avoid to go to the gynaecologist and, in particular, to undertake a cytology? Think about your experience or a relative´s or friend´s experience.
24. Do you think religion could affect the decision about going or not to the gynaecologists? Some people believe that religion does not allow women to be examined by a male doctor, what do you think?
25. How would you like to be invited for a cervical cancer screening? Through a letter / SMS / after an informative event in your community / through a health professional / Others (specify)

CONVERSATIONS AND KNOWLEDGE ABOUT HUMAN PAPILLOMAVIRUS (HPV)

26. Have you ever heard or talked with someone in your social network about the Human Papillomavirus (HPV)? With whom?

Prompts

For example, in your children school?

27. Now that you know what is the cause of the cervical cancer, how likely do you think you would develop cervical cancer? Why?

Prompts

Do you think the religion is a protecting factors against these diseases in your community? Why?

For example, do you think men in your communities could have sex before marriage?

28. Would you do this test to detect this sexually transmitted virus (HPV) or do you think it is not relevant or it would not be accepted in your community?

SCREENING PREFERENCES AND HPV SELF-SAMPLING ACCEPTABILITY

SHORT DEMONSTRATION OF HPV SELF-SAMPLING DEVICES

We are finishing this interview with a few more questions about this device. This is a HPV self-sampling (show instructions, illustrations). Now women have the possibility of taking a vaginal smear themselves at home for cervical cancer screening. This self-sampling device, in particular, is to take a vaginal smear that allows to detect the HPV, which is the necessary cause of cervical cancer.

29. If you would be offered to be screened for cervical cancer, what would you prefer: to go to a clinician or to do it yourself at home? Why?
30. In your opinion, what do you think are the advantages and disadvantages of using this device?
31. If you would decide to use it, how confident would you feel to use it?
    - I would be worried about use it correctly ☐
    - I would feel confident with appropriate instructions ☐
32. Do you think this device (self-sampling) is the solution for those women who do not go to the gynaecologist because of fear, shyness or do not want to be examined by a male doctor?
33. And if you opt to use the self-sampling device, where would you like to receive and return this device? Why? (In a health centre /receive it at home and return to the pharmacy / receive and return it through a community agent)
34. What about an information campaign about cervical cancer screening? How would you like this to be presented? (In a community event with other women / through a video / with illustrations / other ways)
35. Would you like and would you feel yourself confident to become a 'champion' or inform other women in your community about cervical cancer screening?

Thank you for your time!
